# Sexual function in adult patients who have undergone augmentation surgery in childhood: what is really important?

**DOI:** 10.1038/s41443-020-00355-x

**Published:** 2020-10-10

**Authors:** Beatriz Bañuelos Marco, Manuela Hiess, Raimund Stein, Ricardo Gonzalez, Anja Lingnau, Dan Wood, Anna Radford, Bernhard Haid, on behalf of the Pediatric Urology Group of the EAU Young Academic Urologists

**Affiliations:** 1 Department of Urology, Charité Medical University of Berlin, Berlin, Germany; 2 Department of Urology, Medical University of Vienna, Vienna, Austria; 3 Department of Pediatric, Adolescent and Reconstructive Urology, Medical Faculty Mannheim, University of Medical Center Mannheim, Mannheim, Germany; 4 Pediatric Surgery and Urology, Auf der Bult Kinder und Jugend Krankenhaus Hannover, Hannover, Germany; 5 University College London Hospitals NHS Trust, London, United Kingdom; 6 Department of Paediatric Urology, Leeds Teaching Hospitals NHS Trust, Leeds, United Kingdom; 7 Department of Pediatric Urology, Hospital of the Sisters of Charity, Linz, Austria

**Keywords:** Diseases, Reproductive signs and symptoms

## Abstract

Problems relating to the development of a healthy approach to sex and intimacy during puberty, after augmentation cystoplasty, are scarcely discussed in literature. Therefore, this may suggest that such issues are insufficiently addressed by pediatric urologists. We gathered four experts in the field as well as an experienced leader of a patient group and mother of a girl with spina bifida and asked questions relevant to the following areas of care: (a) diversion, urinary incontinence, and sexual life; (b) impact of a stoma on body image perception and self-esteem; (c) specific female concerns with regard to fertility and recurrent urinary infections; (d) specific male concerns on anejaculation and erectile dysfunction. Their answers are discussed in view of the available literature. All experts and the patient group representative agreed that most of these patients will experience: lack of self-confidence as the most frequent obstacle to starting a relationship and incontinence as a barrier to sexual activity. The cosmesis of the stoma and abdominal scars might influence self-esteem and therefore the sexual activity, however it appears to be a less common concern in males than females. Our results outline the importance and influence that the body image, self-esteem, and confidence present for the individual expectations of the patients related to sex life and sexual activity. Physicians should be encouraged to ask all postpubertal patients about their sexual concerns at every visit. Further studies and exchange of information between clinicians are needed to provide meaningful and analyzable patient-related outcome measures (PROMs).

## Introduction

Spina bifida (SB)—the incomplete closure of the neural tube at ~26 days of gestation, occurs in 3.5 per 1000 live births worldwide [1] due to improvement in overall survival, nowadays, >80–95% of children with SB live into adulthood, this improvement in overall survival is likely to be secondary to advances in healthcare [2]. Up to 48% of SB patients with untreated urological problems had evidence of kidney damage [3].

[3] The incidence of renal damage still reaches almost 100% in patients with an overactive pelvic floor (detrusor/sphincter dyssynergia; DSD) who are not properly treated and appears within the first 6 months of life [4]. An early management initiated directly after birth helps convert a high-pressure bladder based on neuropathic detrusor/sphincter dyssynergia into a low-pressure reservoir that is safe for the upper urinary tracts of the SB patients [5]. A simple innovation to help achieve this, is the use of clean intermittent catheterization (CIC) [4].

In a study looking at 104 adult SB patients without previous adequate urological management, a overall rate of 26% of kidney damage on dimercaptosuccinic acid (DMSA) scans was found, its frequency was associated with high leak point pressure (>40 cmH2O), decreased functional bladder capacity as well as detrusor overactivity during [6]. DMSA scans performed in adolescent patients with SB are an accurate tool to assess for renal insufficiency and their results also correlate with arterial hypertension [7].

Bladder and bowel management are part of the main measures towards not only preserving the renal and bladder function but also achieving a better quality of life (QoL) in this group of patients [4]. Maximizing continence care is essential for reaching higher rates of urinary continence, infection-free rates, independency, and a better self-esteem [8].

Augmentation cystoplasty (AC) was developed in the 1950s but not widely used until the advent of the CIC in the 1970s [9] and offers the SB patient a solution to achieve a better continence and preserve the renal function by decreasing the bladder pressure. Its indication is reserved for those patients, where other conservative treatments fail. Especially the advent of intra-detrusor Botulium Toxin (BOTOX) injections has reduced the proportion of patients undergoing these procedures [10]. The SB patients who are selected for this surgery are generally patients with a high bladder pressure, upper tract deterioration and urinary incontinence, which do not respond to intermittent catheterization, oral or intravesical anticholinergic medication and/or intra-detrusor injection therapy with BOTOX.

Problems concerning patients’ sex lives often arise during the critical period of puberty for these patients. When patients transition from pediatric to adult care, the patients and their families face the challenge of leaving a well-trusted environment to an unknown adult urology department or clinic. This is a challenge for the adult urologist who also has to face a difficult process, which is not clearly standardized [11]. Unfortunately there is not enough literature evaluating the QoL of the SB patient [12]. As a consequence, in a survey published in 2006, 75% of young adults with SB did not or could not identify a primary care doctor [13]. While there are enough programs, which guarantee bladder and bowel management, sexual function and fertility are not only a challenge for the physicians but also for the SB patients and their care-takers. This contributes to the emergence of the concept of life-long congenital urology, trying to address the needs of these patients into adulthood in collaboration with “adult” doctors—including a healthy and happy sex life [14].

We have found that patients who have undergone AC and have developed issues around sex during puberty such as the development of sexual desires are scarcely discussed in literature [15]. Therefore the systems currently in place appear to put barriers in the way of discussing such issues with adolescents and it is almost always the responsibility of the caregiver to address such problems. Our aim was to assess the current state of care for adolescent SB patients by approaching four eminent experts and most importantly a patient group representative with questions which was thought to be relevant in daily practice.

The experts were identified by performing a literature review and a group discussion within the pediatric urology expert group of the European Association of Urology Young Academic Urologists. The experts were contacted via email and asked to respond in written form to the questions attached to an invitation letter. The patient group representative was invited to join a telephone interview by one of the authors (BH). She not only offered her personal experience having raised a daughter with SB (currently 20 years old) but is engaged with the leadership of a patient group for over 20 years. She was given adapted questions addressing the same topics as those formulated for the experts. Her answers were transcribed from German to English after thorough discussion to avoid any misunderstanding. Her insights, however, are

Nevertheless a subjective account and representative of one person as well as one cultural background. Whereas theses statements provide a basis for discussion, this limits the conclusions that can be drawn. The initial study protocol (“For patients after AC: what is the ideal content for the transitional consultation in regard to possible future problems.”) was approved by the local ethics committee of the Hospital of the Sisters of Charity, Linz, Austria (Study Nr EKS12/17).

Some of the questions addressed in this paper aim at quantifying the bother or burden of sexual problems related to SB. From a patients’ point of view, there are few but adequate and validated instruments to assess for the QoL and sexual life in SB patients [16, 17]. Reports using the International Index of Erectile Function found a prevalence of erectile dysfunction (ED) of up to 75% [18]. As we are not reporting on patient data, such instruments are not applicable, somewhat limiting the significance of some of the statements herein.

We interviewed: Dr Raimund Stein (RS) Head of Pediatric Urology Department, Universitätsmedizin Mannheim. Dr Dan Wood (DW) Consultant Urologist specialist in Adolescent Reconstruction University College Hospital United Kingdom. Prof. Dr Ricardo Gonzalez (RG), Consultant Pediatric Urologist Auf der Bult-Zentrum für Kinder und Jugendliche, Hannover. Dr Anja Lingnau (AL) Head of Pediatric Urology Department, Charité Universitätsmedizin Berlin.

Silvia Hintringer, Chair of the Austrian Patients Group “Spina Bifida” since over 20 years and mother of a girl with a neurogenic bladder

## Diversion, urinary incontinence, and sexual life

Patients with SB find several impediments in the development of a normal sexuality with self-esteem being one of the most addressed concerns [19]. Urinary incontinence is a proven barrier for a relationship and a healthy sexual life in many urological pathologies [20] correlating with less likelihood of sexual activity [21]. However, little is known about practical aspects as well as the importance of the problem.

### **Questions for the physicians**

What proportion of female/male* patients after augmentation cystoplasty with neurogenic bladder will experience problems related to sexual activities altering/limiting their sexual contacts? Please provide an educated guess based on your experience and a short comment.

RS—First of all, the prevalence of precocious puberty is higher in girls with meningomyelocele compared to the normal population. They do have sexual encounters and around 70% of females can conceive and carry a pregnancy to term. There are only very few data available for females after augmentation. In the Global Better Sex Survey [22] a rate of sexual satisfaction improvement was reported in 26.7% (10.9–42.5%), in those under 45 years of age, six women (66.7%) reported an improvement. This clearly shows, how small the cohorts are [6].

In my own experience (Mannheim and Mainz), sexuality is impaired by the neurological status (with all its consequences) and improved by urinary diversion due to the missing urine incontinence.

DW—Yes, I agree. I would add that the presence of hydrocephalus appears to make things worse from the point of view of sexual function. The Indiana group (Szymanski et al.) [23] showed that QoL declined with the degree of urinary incontinence (i.e., more incontinence equated to worse QoL) but any fecal incontinence (regardless of amount) dramatically affected QoL. RG—Currently I can make any evidence-based opinion.AL—There is only very few data available on this topic. Watanabe et al. [24] reported in 18 patients improvement on QoL and also sexual activity by improving self-image, self-esteem, and the ability to cope after urinary tract reconstruction.

*The questions were originally asked separately for male and female patients.

How important is stoma incontinence in female/male* patients with a catheterisable channel in regards to problems in sexual life? Is this a problem you are concerned with in daily practice?

RS—In the literature there is no study (as far as I know). In my personal experience incontinence of the stoma is rare and if it is incontinent it will be corrected. I remember maybe two females in which sexuality improved after getting continent—but again no data available.

DW—I also do not know of any published data relating to this. I also agree that incontinent Mitrofanoff’s are rare. They can usually be corrected. I cannot think of any particular patient examples relating to sexual function and their Mitrofanoff. Although as they grow and age they can become a problem and if they are wheelchair dependent skincare can be an issue—resulting in sores etc. If they are older with incontinence via their stoma reoperating can be an issue and I would imagine (based on day-to-day experience) that this has a marked impact on QoL and sex life.

RG—I think, that as all problems related to incontinence it is important. Fortunately incontinent catheterizable stomas are not common, but when this occurs, it must be corrected.

AL—Generally speaking, if there is incontinence in the stoma it will be corrected. If there is a direct connection between stoma incontinence and problems in sexual life there is no data available that I can think of. Moreno reported that continent urinary diversion in women results in improved self-image, QoL, and enables greater sexual satisfaction [25].

*The questions were originally asked separately for male and female patients.

### Counterpoint: patient representative

*Is sexuality—with regard to the underlying condition and the bladder augmentation—an important concern for you? E.g., as compared to incontinence, mobility?*

Not as much as one could think. The impression is—especially for women—that a stable partnership is more important to a functioning sex life than the fact that there has been a surgical intervention or there is an underlying problem.

Different from females, in males the “shame” toward even a very understanding partner is probably bigger—and the problem is perceived worse. Furthermore, the problem of feeling ashamed and humbled involves a lower probability of such issues being addressed during a urological visit by male patients.

*Is stoma incontinence—in your opinion—a frequent and important problem when it comes to sexual relationships or rather a rare and non-relevant issue? Is this something that is addressed in your clinic visits? Please comment.*

Yes—this is a relevant and important concern. Things like this would probably not be addressed if the physician would not ask from his part. However, female patients are usually able to deal with incontinence—also during sexual activities and given there is a stable relationship, at least better than males.

In this regard, males are more prone/feel more pressure to get help as compared to women and would be more likely to address this—dependent on age and partnership status some men, however, would not address this, therefore it should be a topic anyway.

### Discussion/summary

As previously exposed, SB patients have difficulties developing sexual activity, self- esteem being one of the most prominent concerns. There is a consensus between the literature, the clinicians consulted and the patient group representative that incontinence is an important limitation for the sexual life of the SB patient. However, also from the experts’ point of view most of the evidence is anecdotal. In the study performed by Choi et al. women who did not suffer urinary incontinence had a better sexual function [26] regarding pain and lubrication.

An interesting point, yet not addressed in literature or brought up by the experts is a potential difference in perception concerning the burden caused by incontinence between women and men. This should be addressed in future studies looking into gender diverse aspects of treatment. Based on the clinical experience of the experts consulted, and supported by the literature, we can infer that urinary diversion may improve sex life by offering a solution to urinary incontinence. Watanabe et al. [24] studied the effect of urinary tract reconstruction in the restitution of not only QoL but also sexual activity due to the improvement of body image, self- esteem, and the ability to cope. Body image and satisfaction with urologic management increased in the study published by Moreno et al. [25].

The patient group representative pointed out that female patients placed greater importance on a stable partner for a fulfilling sexual relationship than the severity of the procedures they underwent or urinary incontinence. Unlike men, who place more emphasis on the negative impact of feeling ashamed. It was noted that for SB men it was very frequent to find a partner with a healthcare/nursing background (80%)—completely different from women. With men—having a large burden and a problem with shame sexual problems and concerns are perceived differently.

## Impact of the stoma on perception of body image and self-esteem

Body image—being most likely affected by the status of scars and stomas—as well as self-esteem have an impact on sexual life as previously discussed; therefore we aimed at investigating the potential effects of scars and stoma cosmetics in SB patients.

### **Questions for the physicians**

*Are the female patients concerned with the cosmetic appearance of their scars or stoma when it comes to sexual relationships? Is this a reason for special considerations during surgery concerning stoma placement? Did you ever indicate re-do surgery because of such concerns?*

RS—No it is sometimes the midline scar after augmentation which looks not so good in obese patients, but for the patient it is usually ok. The scars of the VP shunt are usually more disturbing. So far I did not do a revision due to this reason.

DW—I agree. I have seen this as more of a concern in other groups (e.g., exstrophy) but not in this group and I have not revised any scar.

RG—Yes. Most patients preferred concealed stomas (i.e.,umbilical). I have operated a number of females (and some males) because they were concerned about the appearance of a stoma.

AL—In my experience the functional aspect of the stoma is more important to the patient than the cosmetic appearance. Nevertheless we aim for the umbilicus as exit point and we do the surgery via Pfannenstiel incision if possible.

*How important are cosmetic aspects of the stoma/the abdominal wound in relation to sexual relationships of the male patients? Are the patients concerned with the cosmetic appearance of their scars/their stoma when it comes to sexual relationships?*

RS—Less in males. Usually it goes to the umbilicus.

DW—Agreed.

RG—Yes. Most patients preferred concealed stomas (i.e., umbilical). I have operated a number of females (and some males) because they were concerned about the appearance of a stoma.

AL—In my experience scars are less important in the male cohort than in the female. Also the positioning of the scar is addressed fewer.

*What do you think about the idea that in boys/young men after augmentation cystoplasty, a transumbilical/abdominal stoma for catheterizing is of advantage, because thereby urinary drainage is uncoupled from the genital organs?*

RS—I think it makes no difference, but should be considered.

DW—I agree with Raimund, although I can think of at least two patients who specifically asked for their to not go to their umbilicus as they did not want to touch their umbilicus. I offer them the choice.

RG—It might be true, although I do not know of any evidence to support this assumption.

AL—Strange idea, since the natural urinary outlet is via the genital organs, but of course in spontaneous voiding there is less manipulation

### Counterpoint: patient representative

Are the scars and the cosmetic appearance of the stoma and the abdomen in general an important issue? Is this something that is addressed in your clinic visits?

Other than the above-mentioned issues—scars are much more problematic for female patients. Also the fear about new scars is bigger in female patients. Scars and cosmetic appearance—anyway—are considered more important than e.g., incontinence.

For men—not much of a problem—not only in sexual life but also in daily proceedings. If so, men would be more likely than women to address this.

### Discussion

Negative self-perception has been frequently cited as an obstacle for a satisfying sex life [27].

It can be then inferred that the cosmesis of the stoma may influence the self-esteem and therefore the sexual activity of the patients, this should be taken into consideration as an important issue to address when discussing concerns regarding sex life. Lack of self-confidence is one the most frequent obstacles to starting a relationship. As already mentioned the cosmesis of the stoma might influence the self-esteem and therefore the sex life of the patients [19]. Based on the experience of the consulted experts and the patient representative, it appears to be a less common concern in males as in females. However, this must not be an impediment to address this matter with our patients, as self-esteem and the idea of their own sex lives and sexual satisfaction are based on individual expectations and variations between them.

## Specific female concerns on fertility and recurrent urinary infections

Fertility might be impaired in women with SB, and antenatal complications, fetal loss as well as neural tube defect in there offspring are more frequent, requiring close obstetric and urological surveillance as well as awareness of the importance of folic acid prophylaxis before conception. Patients should be counseled about theses risks before embarking on pregnancy, and when pregnant, they should be managed in a unit, which can provide high- risk obstetric, and urology cover [28].

*As fertility can be considered normal in these patients, is contraception an important issue to consider when talking to adolescent girls after AC? How and when do you address these issues? Do you refer the patients to a (specialized) gynecologist.*

RS—Both recommend to have a regular check-up with their gynecologist—recommend to take an oral contraceptive pill pills (according to the gynecologist recommendations) and use latex-free condoms, which is a problem as they are expensive.

DW—Yes contraception is important. This would usually be directed by their general practitioner (GP) in the United Kingdom but is an important conversation for us to have. Also very important to teach them about the use of folic acid for 3 months before conception and for the first trimester (to reduce their risk of having an affected child). They also need to know that the urine pregnancy test has a 57% false positive rate in anyone who has a cystoplasty (so diagnosis of pregnancy requires measurement of serum human chorionic gonadotropin (HCG).

RG—The girls should be referred to a gynecologist who is informed and can address the questions of the patients.

AL—Contraception should be addressed in puberty and adapted to the individual wishes and needs (e.g., oral contraception or latex-free condoms) of the patients. Regular appointments at a gynecologist should be part of the schedule.

*In young female adult patients who suffer from recurrent urinary tract infections (UTIs): do you regularly address this issue? How important—from your point of view—are topical intimate hygiene after intercourse, CIC and eventually antibiotic prophylactic treatment after intercourse in this group of patients?*

RS—If this is a problem, even in a female after bladder neck closure (*n* = 1), I recommend the same as in the normal population. Additional to do a CIC after sexual intercourse.

DW—Yes this can be a problem and we discuss it a lot. As with Raimund they should undertake all usual measures. Good fluid intake, regular emptying, CIC after intercourse. I suggest they try D-mannose and probiotics (despite lack of evidence), I recommend they washout their bladder once per day (as per Doug Hussmann’s data [29] In some cases more extreme measures such as overnight catheterization might be important. If infections are recurrent—I scope them.

RG—I cannot give an answer based on evidence but intimate hygiene seems important and I have recommended one dose prophylaxis after intercourse.

AL—We address this issue on a regularly basis: CIC after intercourse and if necessary antibiotic prophylactic treatment is recommended. Also a good functioning bowel management helps to avoid UTIs related to intercourse.

### Counterpoint: patient representative

*Are you worried about fertility? Did you get sufficient information about contraception from your (pediatric) urologist? Or do you consult with a gynecologist?*

This is a big issue for every women or girl concerned. It should be routinely addressed by (pediatric) urologists. Gynecologists in the vast experience of the patient representative over 20 years—usually feel not competent to counsel patients with neurogenic bladder emptying disorders and refer them to their urologists. During the urologic visits, the topic often does not get the attention it might deserve.

*Are intercourse-related UTIs an issue in your sexual life? Is this something that is addressed (sufficiently) in your clinic visits?*

Not a real problem even if they would occur: UTIs are part of the daily life and their management is “routine” for these girls.

### Discussion

Of course, the patients group representatives’ opinion that pediatric urologists should cover fertility related issues is a misconception resulting from their personal experience with gynecologists unfamiliar with congenital malformations. The lack of availability of high-quality sex education for patients with SB is also highlighted in the

literature, warranting the establishment of “transitional gynecology in order to properly address these girls needs [15]

Recurrent UTIs are a topic frequently addressed in the regular consultation of SB patients and even more important after urinary diversion and AC due to the anatomical characteristics. UTIs related to intercourse in women are a common presenting complaint There is a lack of evidence relating to treating intercourse-related UTIs in SB patients and therefore it is assumed UTIs should be managed in these patients as in patients with non-neurogenic bladder emptying disorders.

## Specific male concerns on anejaculation and/or erectile dysfunction

Men with SB may also demonstrate orgasm and ejaculatory dysfunction, which has been reported in 75% of affected patients. Quality of ejaculation also appears to be impaired. It has been reported that 78% of men with SB were able to ejaculate, but only 17% reported it to be forceful. Like erectile quality, ejaculatory function also correlates with the level of the lesion [30, 31]. Men with SB also ejaculate less frequently than the general population, and ejaculation can occur differently, often being described as more of a dribble than propulsive. In view of these data we aimed at looking into an experts and patient representatives perspective of theses specific problems.

*In patients with thoracic or high lumbar lesions: is anejaculation important—in terms of “causing them trouble” with their sexual relationships or is this a minor issue in your perception? Please comment.*

RS—More a minor problem—often reported, but so far no one who really like to have a child.

DW—It is described and is a concern for fertility, treatments such as electro-ejaculation may help. My impression (without evidence) is that this is more of a concern to the Spinal Cord Injury group rather than the myelomeningocele.

RG—I cannot say. Patients with anejeculation who wish to be fertile should be referred to fertility specialists.

AL—We referred them to the fertility specialist and andrology specialist.

*Generally, should ED be part of the counseling in patients after AC based on a neurogenic bladder before age 18? Is there a basis to consider ED a prevalent problem in this group of patients? Do you do this yourself or would you suggest referring them?*

RS—I talk to them before during and after puberty, sometimes prescribe sildenafil, sometimes recommend a visit with a sexual therapist.

DW—Important conversations to have as Raimund have suggested and important to revisit. If Phosphodiesterase-5 inhibitors fail I would refer to our andrology team.

RG—Yes, yes. I refer them to a specialist.

AL—We would refer the patient to a colleague specialized in ED.

### Counterpoint: patient representative

*Is anejaculation (i.e., no semen liquid coming out)—in terms of “causing them trouble” with their sexual relationships or is this a minor issue in your perception? Please comment.*

Rather a big issue and unlikely to be mentioned by the boys/men by themselves. It should be addressed without initiative from the men—and boys (referring to masturbation)/who often feel ashamed to ask. And it should be already probably done at an earlier age, maybe around 14 years old.

*Are you worried about ED (i.e., not being able to achieve a sufficient erection of the penis allowing for penetration)?*

Same as above. It should be addressed without initiative from the men and boys at an earlier age.

### Discussion/summary

Over 91% of patients indicate that physicians should talk to patients with SB about their sexual health. However, more than half of those patients with SB have no recollection of such a conversation with their providers [18]. This is addressed by the patient group representative in the interview, whereas the experts consulted referred the patients mostly to a specialist (andrologist). Both patient and experts agreed that they bring the topic up with the patients once in puberty.

The treatment of ED in SB patients is possible and effective [32]. It is highly important for a healthy and happy sex life, self-confidence, and maintaining long-term relationships. Men with ED often respond to established therapies, including oral medications.

People with SB experience sensation differently, with “normal” sensation reported in only 20% of cases [33]. In men, only 41% have normal erections, with ambulant men more likely to report normal erections than those in a wheelchair. Additionally, those with SB are less likely to have feelings of sexual excitement consistent with orgasm compared to the general population. An innovative method of enhancing sensation with regards to sexuality is the TOMAX procedure, involving a microsurgical connection between the ilioinguinal nerve and the dorsal penile nerve. While it clearly requires a special set of expertise it has been shown to be effective in a relevant proportion of patients [34].

Regarding fertility, subfertility is common concern, despite the fact that testosterone levels have been reported to be normal, there seems to be a failure on the level of Sertoli Cells or germinal cells [30, 8].

## Limitations

We need to highlight/understand as a limitation the way how these questions were conceived. After reviewing the literature and the experience of the authors in a structured discussion within the Pediatric Urology Expert Group of the Young Academic Urologists we identified the field we wanted to explore and drafted the questions after repeated discussion in the group before sending them to the experts. We feel that the topic we addressed in this article is very important for these patients and the clinicians treating them.

## Conclusion

The various difficulties and impediments, SB patients developing sexual activity have to face, are poorly reported on in literature—especially concerning information that can be readily included into counseling these patients. Self-esteem was one of the most important concerns and urinary incontinence was identified as a limitation. Therefore, urinary diversion may improve sexual life by offering a solution to urinary incontinence. Female patients found important a stable partner for a fulfilling sexual relationship, whereas men stress more the fact of feeling ashamed or pressured by urinary incontinence. Recurrent UTIs and those related to intercourse in women are a usual urological consultation and frequently addressed in the regular consultation of SB patients, especially after urinary diversion.

Patients and their representatives express their concern on the lack of sexual education based on their personal experience with gynecologists unfamiliar with congenital malformations, and this is supported by the available literature [15]. Physicians should be encouraged to ask all postpubertal patients if they have any urinary, fecal, or sexual concerns at every visit to both establish a solid physician–patient relationship, which helps to facilitate conversations about sex and also confer the trust on the consult as a safe place to express doubts and concerns regarding their sex lives. Patients with SB find impediments to develop normal sexual relationships for a variety of reasons, including impaired self-esteem and dependence on caregivers.

We do not intend to conclude evidence-based recommendations but to raise awareness to a very important topic that currently is scarcely documented in the literature. Therefore, our conclusions are based on the opinion of experts respected on their field and should be not standardized in the clinical practice, but considered for further study.
