# Correction to: Sexual function in adult patients who have undergone augmentation surgery in childhood: what is really important?

**DOI:** 10.1038/s41443-021-00500-0

**Published:** 2021-12-01

**Authors:** Beatriz Bañuelos Marco, Manuela Hiess, Raimund Stein, Ricardo Gonzalez, Anja Lingnau, Dan Wood, Anna Radford, Bernhard Haid, on behalf of the Pediatric Urology Group of the EAU Young Academic Urologists

**Affiliations:** 1 Department of Urology, Charité Medical University of Berlin, Berlin, Germany; 2 Department of Urology, Medical University of Vienna, Vienna, Austria; 3 Department of Pediatric, Adolescent and Reconstructive Urology, Medical Faculty Mannheim, University of Medical Center Mannheim, Mannheim, Germany; 4 Pediatric Surgery and Urology, Auf der Bult Kinder und Jugend Krankenhaus Hannover, Hannover, Germany; 5 University College London Hospitals NHS Trust, London, UK; 6 Department of Paediatric Urology, Leeds Teaching Hospitals NHS Trust, Leeds, UK; 7 Department of Pediatric Urology, Hospital of the Sisters of Charity, Linz, Austria

**Keywords:** Diseases, Reproductive signs and symptoms

Correction to: *International Journal of Impotence Research* 10.1038/s41443-020-00355-x, published online 10 October 2020

The article Sexual function in adult patients who have undergone augmentation surgery in childhood: what is really important?, written by Beatriz Bañuelos Marco, Manuela Hiess, Raimund Stein, Ricardo Gonzalez, Anja Lingnau, Dan Wood, Anna Radford, Bernhard Haid, on behalf of the Pediatric Urology Group of the EAU Young Academic Urologists, was originally published Online First without Open Access. After publication in volume 33, issue 2, page 170–177 the author decided to opt for Open Choice and to make the article an Open Access publication. Therefore, the copyright of the article has been changed to © The Author(s) 2020 and the article is forthwith distributed under the terms of the Creative Commons Attribution 4.0 International License, which permits use, sharing, adaptation, distribution and reproduction in any medium or format, as long as you give appropriate credit to the original author(s) and the source, provide a link to the Creative Commons licence, and indicate if changes were made. The images or other third party material in this article are included in the article’s Creative Commons licence, unless indicated otherwise in a credit line to the material. If material is not included in the article’s Creative Commons licence and your intended use is not permitted by statutory regulation or exceeds the permitted use, you will need to obtain permission directly from the copyright holder. To view a copy of this licence, visit http://creativecommons.org/licenses/by/4.0.

Open access funding enabled and organized by Projekt DEAL.

